# Development of a Machine Learning-Based Model for Predicting the Incidence of Peripheral Intravenous Catheter-Associated Phlebitis

**DOI:** 10.2478/jccm-2024-0028

**Published:** 2024-07-31

**Authors:** Hideto Yasuda, Claire M. Rickard, Olivier Mimoz, Nicole Marsh, Jessica A Schults, Bertrand Drugeon, Masahiro Kashiura, Yuki Kishihara, Yutaro Shinzato, Midori Koike, Takashi Moriya, Yuki Kotani, Natsuki Kondo, Kosuke Sekine, Nobuaki Shime, Keita Morikane, Takayuki Abe

**Affiliations:** Department of Emergency and Critical Care Medicine, Jichi Medical University Saimata Medical Center, Saitama, Japan; Department of Clinical Research Education and Training Unit, Keio University Hospital Clinical and Translational Research Center (CTR), Tokyo, Japan; School of Nursing, Midwifery and Social Work, UQ Centre for Clinical Research, The University of Queensland, QLD, Australia; School of Nursing and Midwifery; Alliance for Vascular Access Teaching and Research, Griffith University, Nathan, QLD, Australia; Herston Infectious Diseases Institute; Nursing and Midwifery Research Centre, Royal Brisbane and Women's Hospital, Metro North Health, Herston, QLD, Australia; CHU de Poitiers, Emergency Department and prehospital care, Poitiers, France; University of Poitiers, INSERM U1070, Pharmacology of antimicrobial agents and antibiotics resistance (PHAR2), Poitiers, France; University of Poitiers, Faculty of Medicine and Pharmacy, Poitiers, France; Department of Intensive Care Medicine, Kameda Medical Center, Chiba, Japan; Department of Emergency Medicine, Koga Community Hospital, Shizuoka, Japan; Department of Medical Engineer, Kameda Medical Center, Chiba, Japan; Department of Emergency and Critical Care Medicine, Graduate School of Biomedical and Health Sciences, Hiroshima University, Hiroshima, Japan; Division of Clinical Laboratory and Infection Control, Yamagata University Hospital, Yamagata, Japan; Biostatistics, Clinical and Translational Research Center, Keio University School of Medicine, Tokyo, Japan; School of Data Science, Kyoto Women’s University, Kyoto, Japan

**Keywords:** phlebitis, complication, machine learning, prediction model, risk factor

## Abstract

**Introduction:**

Early and accurate identification of high-risk patients with peripheral intravascular catheter (PIVC)-related phlebitis is vital to prevent medical device-related complications.

**Aim of the study:**

This study aimed to develop and validate a machine learning-based model for predicting the incidence of PIVC-related phlebitis in critically ill patients.

**Materials and methods:**

Four machine learning models were created using data from patients ≥ 18 years with a newly inserted PIVC during intensive care unit admission. Models were developed and validated using a 7:3 split. Random survival forest (RSF) was used to create predictive models for time-to-event outcomes. Logistic regression with least absolute reduction and selection operator (LASSO), random forest (RF), and gradient boosting decision tree were used to develop predictive models that treat outcome as a binary variable. Cox proportional hazards (COX) and logistic regression (LR) were used as comparators for time-to-event and binary outcomes, respectively.

**Results:**

The final cohort had 3429 PIVCs, which were divided into the development cohort (2400 PIVCs) and validation cohort (1029 PIVCs). The c-statistic (95% confidence interval) of the models in the validation cohort for discrimination were as follows: RSF, 0.689 (0.627–0.750); LASSO, 0.664 (0.610–0.717); RF, 0.699 (0.645–0.753); gradient boosting tree, 0.699 (0.647–0.750); COX, 0.516 (0.454–0.578); and LR, 0.633 (0.575–0.691). No significant difference was observed among the c-statistic of the four models for binary outcome. However, RSF had a higher c-statistic than COX. The important predictive factors in RSF included inserted site, catheter material, age, and nicardipine, whereas those in RF included catheter dwell duration, nicardipine, and age.

**Conclusions:**

The RSF model for the survival time analysis of phlebitis occurrence showed relatively high prediction performance compared with the COX model. No significant differences in prediction performance were observed among the models with phlebitis occurrence as the binary outcome.

## Introduction

Peripheral intravenous catheters (PIVCs) are the most commonly used invasive medical devices in hospitalised patients, especially critically ill patients in intensive care units (ICUs) [1]. However, PIVCs are associated with different types of complications, with phlebitis being the most common [1–2]. PIVC-associated phlebitis is a serious healthcare complication because it not only causes patient discomfort but can also result in infections or other outcomes, thus resulting in prolonged hospital stays and increased expenditure [3,4,5,6,7,8,9,10,11,12,13,14]. The early identification and accurate assessment of PIVCs is essential to prevent the occurrence of PIVC-associated phlebitis. Machine learning techniques have been established as a reliable and robust tool to predict outcomes in emergency and critical care settings [15,16,17,18,19,20,21,22]. Implementing machine learning (ML)-based predictive modelling in ICUs with electronic medical records could improve healthcare quality by alerting medical staff of impending complications in advance, and the timely removal of symptomatic PIVCs is important to prevent the occurrence of phlebitis [5]. The Infusion Nurses Society and National Health Service hospitals in England (epic3) do not recommend the routine replacement of PIVCs but recommend replacement if clinical findings of suspected infection, such as phlebitis, or other complications are observed [23,24]. However, given that phlebitis has resulted in a need for skin grafting, progressed to necrosis, and increased the risk of death in severe cases [11], a predictive model for phlebitis would aid in the removal of PIVCs when clinically indicated rather than at predesignated time periods. Although several studies have investigated the risk factors for the occurrence of phlebitis, few models have been developed to predict PIVC-associated phlebitis [25]. To bridge this knowledge gap, this study utilised the AMOR-VENUS database, which is an epidemiological database of PIVCs in critically ill patients [1]. The current study aimed to develop several ML-based models by using previously identified risk factors to predict the occurrence of PIVC-associated phlebitis [1,14] and to validate and compare the predictive performance of these models.

## Materials and methods

### Study design and setting

This study used the AMOR-VENUS database [1], a prospective multicentre cohort study in 23 ICUs of 22 institutions that was conducted in Japan between January 1, 2018, and March 31, 2018. The AMOR-VENUS study was conducted to describe the epidemiology of PIVC use and the incidence/occurrence of phlebitis and related complications in the ICU.

### Ethical considerations

Post-hoc analyses using the AMOR-VENUS database were approved by the AMOR-VENUS ethics review board. Therefore, the requirement for a new ethics review for this study was waived. The study was conducted in accordance with the Declaration of Helsinki and Transparent Reporting of Multivariable Prediction Model for Individual Prognosis or Diagnosis Statement [26]. The need for informed consent was waived, and an opt-out recruitment method was employed.

#### Study participants and included PIVCs

This study included data from the AMOR-VENUS database on all consecutive newly inserted PIVCs in patients ≥ 18 years during their ICU stay. The inclusion and exclusion criteria can be found in a previously published article on the AMOR-VENUS study [1]. These PIVCs were randomly divided into a development cohort and a validation cohort in a 7:3 ratio to improve the prediction accuracy of the development cohort. The following selections were left to the discretion of the physicians at each study institution: catheter type (material and catheter gauge), medical staff for PIVC insertion, insertion method (insertion site, antiseptic solution, use of ultrasound, and glove type), management method (type of dressing and timing of changing the dressing), and removal timing.

### Data collection

The following variables were retrieved from the AMOR-VENUS study database: ICU characteristics (provision of standard ICU drug administration procedures, education of nurses handling the intravenous [IV] catheter management); patient characteristics (age, sex, height, weight, acute physiological and chronic health evaluation [APACHE] II score [27], Charlson comorbidity index [28], ICU admission route, mode of admission, admission category, and admission with presence of sepsis [29]); characteristics of PIVC (medical staff inserting the catheter, insertion site, catheter material, catheter gauge, dressing method, infection during catheter indwell, and duration of catheter indwell); drugs administered via PIVC (drug concentration, administration rate and duration of administration); and phlebitis-related outcomes. Details on the data collected in the original article can be found in Appendix File A.

### Outcomes

The occurrence of phlebitis was considered the primary endpoint, which was defined using the phlebitis scale developed by the Infusion Nurses Society [30]. Detailed information regarding the definition of phlebitis and assessment methods has been reported in the AMOR-VENUS study [1].

### Predictors of outcome

Forty potential variables from four variable levels were included in the prediction models and were selected on the basis of a priori knowledge [3,4,5,6,7,8,9,10,11,12,13,14] and clinical perspectives. These included ICU characteristics (education of nurses regarding venous catheter management and standardised drug administration measures in the ICU); patient-level variables (age, sex, body mass index, APACHE II score, and presence of infections during catheter dwelling); catheter-level variables (designation of medical staff inserting the catheter, number of insertion attempts, use of ultrasonography, catheter insertion site, catheter gauge, type of dressing, and catheter material); and drug-level variables (fentanyl, heparin, propofol, nicardipine, dexmedetomidine, ampicillin/sulbactam, albumin, paracetamol, potassium, meropenem, steroid, ceftriaxone, vancomycin, magnesium, peripheral parenteral nutrition, phosphorus, noradrenaline, carperitide, midazolam, nitroglycerin, dobutamine, cefmetazole, amiodarone, cefepime, levetiracetam, and landiolol). The types of drugs included in the prediction model were limited to those administered via more than 1% of all PIVCs and were associated with a phlebitis incidence of more than 5% on the basis of clinical considerations. The standardised drug administration measures in the ICU in the current study were defined in accordance with the documented standard operating procedures for drug administration, which was supervised by a pharmacist at the institution, and included the drug’s composition, choice of route of administration, administration rate, and contraindications to compounding.

### Data processing

Spline curves were drawn to evaluate whether the continuous variables (age, body mass index, and APACHE II) had a linear effect on the occurrence of phlebitis. If the effect was not linear, cutoff values were set in accordance with the spline curves and were treated as categorical variables. Drugs were included in the prediction model as binary data. Catheter gauges were categorised into 14–16, 18, 20, and 22–24 gauges. Dressing was divided into sterile or non-sterile. Other factors as registered in the database were included in the prediction model. Continuous variables were standardised, and categorical variables were transformed into dummy variables.

### Handling of missing data

Variables with >30% of missing data were excluded. For continuous variables, outliers and apparently inconsistent values were treated as missing. However, none of the included variables had a missing percentage > 30%, outliers, and apparently inconsistent value. Multiple imputation was performed using the “mice” package to impute missing values (m = 25, maxit = 50, method = ‘pmm’, seed = 500) [31]. Missing measures were imputed using all predictors, outcomes, and other covariates. Details on the missing imputation method have been provided in Appendix File A.

### Sample size calculation

According to the criteria of Riley et al. [32], a total of 1429 participants (PIVCs) were needed in both the development and validation cohorts to determine a shrinkage of <10%, estimated prevalence of 7.5% for PIVC-related phlebitis [1], and expected R-squared value of 0.2 by using 40 potential predictive parameters.

### Statistical analysis

#### Patient characteristics and predictors

Patient characteristics, catheter characteristics, and candidate predictors for each cohort were described as mean and standard deviation (SD) or median and interquartile range (IQR) for continuous variables and as numbers and percentages for categorical variables.

#### Machine learning models

The included PIVCs were randomly divided into a development cohort and a validation cohort in a 7:3 ratio. This ratio was chosen based on a similar study that used machine learning for predictive modeling in the emergency department setting [33], which also employed a 7:3 split. The development cohort was used to train and optimize the machine learning models, while the validation cohort was used to assess the performance of the developed models on unseen data. Given that the time to occurrence of phlebitis is an important outcome for PIVC-related phlebitis, we used the random survival forest (RSF), which can create predictive models for time-to-event outcomes, for the development cohort and devised a predictive model [34]. Moreover, considering that the occurrence of phlebitis is also an important outcome, the (1) logistic regression (LR) with least absolute reduction and selection operator (LASSO), (2) random forest (RF), and (3) gradient boosting decision tree were chosen to develop predictive models that treat outcome as a binary variable [15,16,17,18,19,20,21,22]. RSF models were built using the R package randomForestSRC, and the variable importance was obtained [35]. To understand the contribution of the predictors to the model, a scaled variable importance of 100 was shown as the maximum value [36, 37]. LASSO was performed to select the optimal value of penalty parameter (lambda, λ). Validation was performed using 10-fold cross-validation, and beta coefficients for the selected variables were calculated. For the development of RF and gradient-boosted tree models, we performed hyperparameter optimisation with a grid search strategy by using the “ranger” and “caret” packages [36, 38]. For the gradient-boosted tree model, we used 10-fold cross-validation to measure the prediction error with a smaller variance than the prediction error from a single train-test split. Similarly, the RSF and RF models measured prediction error by using out-of-bag (samples left behind after bagging) estimation.

#### Comparator models

To compare the predictive performance, we chose the Cox proportional hazards (COX) and LR models as comparators for time-to-event and binary outcomes, respectively. Backward stepwise selection methods were used to determine the optimal factors for COX and LR models. To ensure consistency and comparability in the analysis, the risk factors for phlebitis used in these models were the same as those used in the abovementioned four ML models. Details on the comparator models have been provided in Appendix File A.

#### Assessment of model performance

To assess the predictive performance, the developed models, including the COX and LR comparator models, were applied to the validation cohort. Receiver operating characteristic (ROC) curves were drawn, and c-statistics (also called area under the curve [AUC]) with 95% confidence interval (CI) were calculated as the discriminant index. In addition, the c-statistics of the models were compared using the Delong test for each outcome type [39]. To show the relationship between the predicted and observed probabilities of phlebitis occurrence in the validation cohort, we plotted the calibration curves for the models by using locally weighted scatterplot smoothing curves [40]. All statistical analyses were performed using R software (The R Foundation for Statistical Computing, version 4.0.3) [41].

## Results

### Patients, catheters, and drug characteristics

**Fig. 1.**
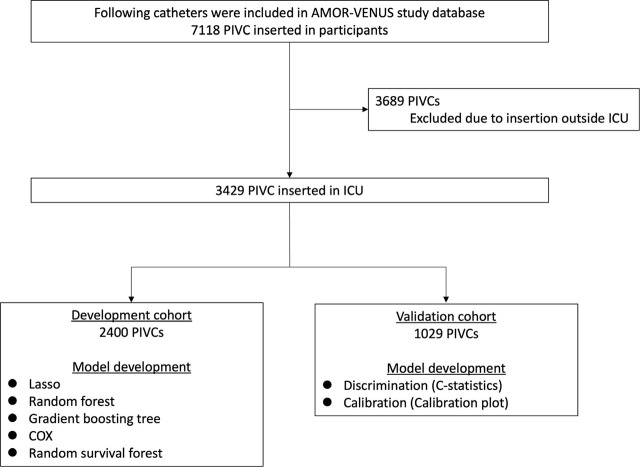
**Patient PIVC flowchart** (ICU, intensive care unit; PIVC, peripheral intravenous catheter)

Among the 7118 PIVCs enrolled in the AMOR-VENUS study, 3689 PIVCs inserted outside the ICU were excluded, thus resulting in a final cohort of 3429 PIVCs (Figure 1). PIVCs were randomly divided into a development cohort (2400 PIVCs) and a validation cohort (1029 PIVCs). Patient and catheter characteristics are shown in Tables 1 and 2, respectively. The median (IQR) catheter dwell time and incidence of phlebitis occurrence in the development cohort were 44.7 (20.7–79.1) h and 208/2400 (8.7%), respectively, and those in the validation cohort were 41.5 (21.0–76.5) h and 105/1029 (10.2%), respectively. The characteristics of drugs administered via PIVC that were included in the ML models are shown in Table A.1 in Appendix File A. Patient, catheter, and drug characteristics for the development and validation cohorts by phlebitis occurrence are shown in Tables A.2, A.3, and A.4 in Appendix File A, respectively. Missing values in the patients and PIVCs are shown in Table A.5 and Table A.6 in Appendix File A. No missing values were found in the context of drugs administered via the PIVCs.

**Table 1.** Patient characteristics of the development and validation cohorts at ICU admission

**Variables**	**Development cohort (N = 2,400)**	**Validation cohort (N = 1,029)**
Age, mean (SD), years	68.1 (15.2)	67.9 (15.0)
Sex, male (n, %)	1485 (61.9)	637 (61.9)
Body height, mean (SD), cm	161 (9.6)	161 (9.6)
Body weight, mean (SD), kg	59.9 (15.4)	60.1 (14.9)
BMI, mean (SD)	23.0 (4.7)	23.1 (4.5)
APACHE II, mean (SD)	19.2 (8.3)	19.2 (8.2)
SAPS II, mean (SD)	44.0 (19.5)	44.3 (19.0)
SOFA, mean (SD)	6.8 (3.7)	6.7 (3.7)
Charlson comorbidity index, mean (SD)	4.2 (2.6)	4.2 (2.6)

ICU admission from (n, %)		
Operation room	921 (38.4)	427 (41.5)
Emergency room	965 (40.2)	404 (39.3)
General ward	363 (15.1)	142 (13.8)
Outpatients	18 (0.8)	2 (0.2)
Transfer from other hospital	133 (5.5)	54 (5.3)

Type of admission to the ICU (n, %)		
Elective surgical	478 (19.9)	214 (20.8)
Emergency surgical	443 (18.5)	213 (20.7)
Medical	1479 (61.6)	602 (58.5)

ICU admission category (n, %)		
Cardiology	860 (35.8)	341 (33.1)
Pulmonary	350 (14.6)	160 (15.6)
Gastrointestinal	243 (10.1)	100 (9.7)
Neurology	455 (19.0)	212 (20.6)
Trauma	95 (4.0)	41 (4.0)
Urology	21 (0.9)	10 (1.0)
Gynaecology	16 (0.7)	8 (0.8)
Skin/tissue	33 (1.4)	17 (1.7)
Others	327 (13.6)	140 (13.6)
Sepsis at ICU admission (n, %)	495 (20.6)	209 (20.3)
Mechanical ventilation within 24 hours after admission to ICU (n, %)	1433 (59.7)	631 (61.3)

APACHE, acute physiology and chronic health evaluation; BMI, body mass index; ICU, intensive care unit; SAPS, simplified acute physiology score; SD, standard deviation; SOFA, sequential organ failure assessment

**Table 2.** PIVC characteristics during the insertion of the development and validation cohorts

**Variables**	**Development cohort (N = 2,400)**	**Validation cohort (N = 1,029)**
Catheter inserted by (n,%)		
Doctor	203/1,879 (10.8)	81/801 (10.1)
Nurse	1,673/1,879 (89.0)	720/801 (89.9)

Inserted Site (n, %)		
Upper arm	245/2,378 (10.3)	111/1,021 (10.9)
Forearm	1,303/2,378 (54.8)	546/1,021 (53.5)
Elbow	113/2,378 4.8)	50/1,021 (4.9)
Wrist	118/2,378 5.0)	44/1,021 (4.3)
Hand	341/2,378 (14.3)	166/1,021 (16.3)
Lower leg	152/2,378 (6.4)	73/1,021 (7.1)
Dorsal foot	106/2,378 (4.5)	31/1,021 (3.0)

Catheter material		
PEU-Vialon*	777/2,400 (32.4)	310/1,029 (30.1)
Polyurethane	658/2,400 (27.4)	320/1,029 (31.1)
Polyethylene	0/2,400 (0)	0/1,029 (0)
Tetrafluoroethylene	910/2,400 (37.9)	382/1,029 (37.1)
Others	55/2,400 (2.3)	17/1,029 (1.7)

Catheter gauge (n,%)		
14G	1/2,357 (0.04%)	0/1,011 (0)
16G	51/2,357 (2.2)	22/1,011 (2.2)
18G	56/2,357 (2.4)	33/1,011 (3.3)
20G	612/2,357 (26.0)	276/1,011 (27.3)
22G	1,592/2,357 (67.5)	662/1,011 (65.5)
24G	45/2,357 (1.9)	17/1,011 (1.7)

Antiseptic solution before catheterisation (n,%)		
None	5/1,863 (0.3)	3/802 (0.4)
Alcohol	1,817/1,863 (97.5)	782/802 (97.5)
0.2% chlorhexidine alcohol	14/1,863 (0.8)	7/802 (0.9)
0.5% chlorhexidine alcohol	10/1,863 (0.5)	5/802 (0.6)
1.0% chlorhexidine alcohol	12/1,863 (0.6)	5/802 (0.6)
10% povidone iodine	2/1,863 (0.1)	0/802 (0)
other	3/1,863 (0.2)	0/802 (0)
Use of ultrasonography (n,%)	36/1,844 (1.9)	22/792 (2.8)

Number of trials for insertion (n,%)		
1	1,501/1,834 (81.8)	618/785 (79.7)
2	221/1,834 (12.1)	92/785 (11.7)
≥3	112/1,834 (6.1)	75/785 (9.6)

Difficulties with the insertions (n, %)		
Easy	882/1,811 (48.7)	350/783 (44.7)
Slightly easy	535/1,811 (29.5)	237/783 (30.3)
Slightly difficult	306/1,811 (16.9)	150/783 (19.2)
Difficult	88/1,811 (4.9)	46/783 (5.9)

Glove (n,%)		
Sterile	14/1,836 (0.8)	5/794 (0.6)
Non-sterile	1,738/1,836 (94.7)	758/794 (95.5)
Nothing	84/1,836 (4.6)	31/794 (3.9)

Dressing (n,%)		
Chlorhexidine-impregnated dressing chrolehexidne	0/2,377 (0)	0/1,019 (0)
Sterile polyurethane dressing	2,338/2,377 (98.4)	989/1,019 (97.1)
Non-sterile polyurethane dressing polyuretane	35/2,377 (1.5)	25/1,019 (2.5)
Gauze dressing	0/2,377 (0)	1/1,019 (0.1)
Tape dressing	4/2,377 (0.2)	4/1,019 (0.4)
Any infection during catheter dwell (n, %)**	550/2,400 (22.9)	253/1,029 (24.6)
Duration of catheter dwell, median (IQR), hour	44.7 (20.7–79.1)	41.5 (21.0–76.5)
Phlebitis (n,%)	208/2,400 (8.7)	105/1,029 (10.2)

ICU, intensive care unit; IQR, interquartile range; PIVC, peripheral intravenous catheter;

* PEU-Vialon: polyetherurethane without leachable additives;

** Any type of infection that the patient had during the period when the target PIVC was inserted

### Predictor selection, model development, and internal validation

**Fig. 2.**
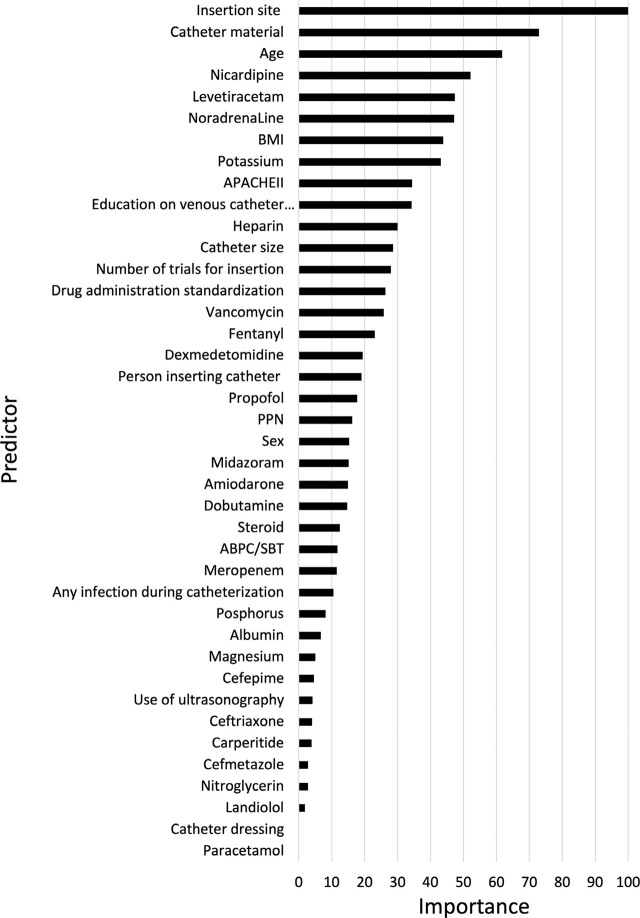
Importance of predictors in the random survival forest model. The variable importance was measured and scaled to have a maximum value of 100.

**Fig. 3.**
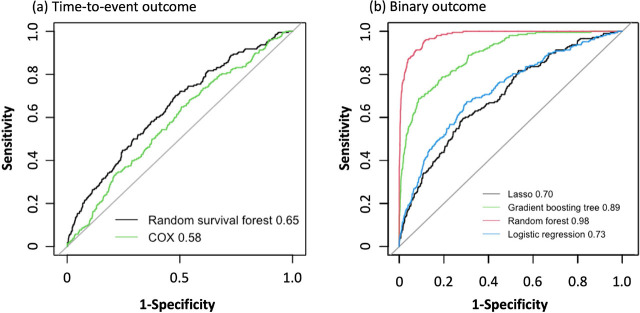
**Receiver operating characteristic (ROC) curves of each model in the development set. (a) The c-statics (95% CI) of the models for time-to-event outcomes; random survival forest: 0.645 (0.606–0.684), and Cox proportional hazards model: 0.581 (0.542–0.621). The black and green lines represent the random survival forest and Cox proportional hazards models, respectively. (b) The c-statics (95% CI) of the models for binary outcomes: LASSO, 0.699 (0.662–0.736); random forest, 0.980 (0.973–0.986); gradient boosting tree, 0.892 (0.870–0.914); and logistic regression, 0.725 (0.688–0.762). The black, red, green, and blue lines represent LASSO, random forest, gradient boosting tree, and logistic regression, respectively.** CI: Confidence interval

The ML prediction models included 40 predictors (59 parameters) for RSF and 41 predictors (60 parameters) for LASSO, RF, and gradient boosting (Table A.7 in Appendix File A). Spline curves of age and body mass index for the occurrence of phlebitis are shown in Figure A.1 in Appendix File A. Age showed a linear association with phlebitis, but body mass index and APACHE II were not considered to be a linear effect on the occurrence of phlebitis. Therefore, the spline curves were used as a comparator to set the cutoff values of body mass index (≤15, 16–22, 23–29, and 30≤), and APACHE II (≤15, 16–25, and 26≤). The importance of predictors in the RSF, RF, and gradient boosting tree models are shown in Figure 2 and Figure A.2 in Appendix File A. A total of 38 predictors (58 parameters), 40 predictors (53 parameters), and 33 predictors (43 parameters) were included in the RSF, RF, and gradient boosting tree models after excluding predictors with zero importance, respectively. In the final LASSO model with optimal λ to minimise the mean squared error, 29 predictors (44 parameters) were selected, the beta coefficient values of which are shown in Figure A.2 in Appendix File A. Factor selection using the backward method for COX and LR models as comparators resulted in the selection of 17 predictors (26 parameters) and 19 predictors (26 parameters), respectively. Hazard and odds ratios and 95% CIs for each model are shown in Figure A.2 in Appendix File A. The other hyperparameters of ML models are described in Table 8 in Appendix File A. Changes in the number of trees and out-of-bag estimation of RF are shown in Figure A.3 in Appendix File A. The internal validation of different models, as represented by ROC curves and c-statistics, are shown in Figure 3. The c-statistic (95% CI) of the comparator models were 0.581 (0.542–0.621) in the COX model and 0.725 (0.688–0.762) in LR model (Table 3). The c-statistic (95% CI) and the results of the Delong test in the development set were 0.645 (0.606–0.688), 0.699 (0.662–0.736), 0.980 (0.973–0.986), and 0.892 (0.870–0.914) in the RSF, LASSO, RF, and gradient boosting tree models, respectively (Table 3 and Table A.9 in Appendix File A).

**Table 3.** Difference of c-statistics in each model in the development cohort

**Model**	**C-statistics (95% CI)**
Binary outcome models	
LASSO	0.699 (0.662–0.736)
Random forest	0.980 (0.973–0.986)
Gradient boosting tree	0.892 (0.870–0.914)
Logistic regression model	0.725 (0.688–0.762)

Survival outcome models	
Random survival forest	0.645 (0.606–0.684)
Cox proportional hazards model	0.581 (0.542–0.621)

CI: Confidence interval

### Model performance in validation cohort

**Fig. 4.**
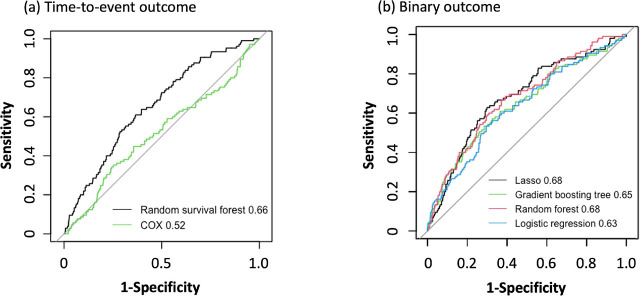
**Receiver operating characteristic (ROC) curves of each model in the validation set. (a) The c-statics (95% CI) of the models for time-to-event outcomes: random survival forest, 0.655 (0.603–0.708); Cox Proportional Hazard Model, 0.516 (0.454–0.578). The black and green lines represent the random survival forest and Cox proportional hazards models, respectively. (b) The c-statics (95% CI) of the models for binary outcomes: LASSO, 0.680 (0.625–0.735); random forest, 0.677 (0.622–0.731); gradient boosting tree, 0.646 (0.587–0.706); and logistic regression, 0.633 (0.575–0.691). The black, red, green, and blue lines represent LASSO, random forest, gradient boosting tree, and logistic regression, respectively.** CI: Confidence interval

The c-statistics (95% CI) of the models in validation cohort for discrimination were as follows: RSF, 0.689 (0.627–0.750); LASSO, 0.664 (0.610–0.717); RF, 0.699 (0.645–0.753); gradient boosting tree, 0.699 (0.647–0.750); COX, 0.516 (0.454–0.578); and LR, 0.633 (0.575–0.691) (Table 4). The results of the Delong test are shown in Table A.10 in Appendix File A. The plotted ROCs are depicted in Figure 4. No significant difference was observed among the c-statistic (95% CI) of the three models in terms of binary outcome (LASSO, RF, and gradient boosting). For the visual assessment of calibration plot in the validation cohort (Figure A.4 in Appendix File A), all four ML models were well calibrated to the observed overall range of predicted phlebitis in the low range of the predicted outcome. The Brier scores of the models for binary outcomes and the RSF model for time-to-event outcome were 0.089 and 0.113, respectively (Table A.11 in Appendix File A). Other indicators of model performance in the predictive model that treated outcomes as binary variables are shown in Table A.12 in Appendix File A.

**Table 4.** Difference of c-statistics in each model in the validation cohort

**Model**	**C-statistics (95%CI)**
Binary outcome models	
LASSO	0.680 (0.625–0.735)
Random forest	0.677 (0.622–0.731)
Gradient boosting tree	0.646 (0.587–0.706)
Logistic regression model	0.633 (0.575–0.691)

Survival outcome models	
Random survival forest	0.655 (0.603–0.708)
Cox proportional hazards model	0.516 (0.454–0.578)

CI: Confidence interval

## Discussion

The RSF model for the survival time analysis of phlebitis occurrence showed relatively high prediction performance compared with the COX model. However, no significant differences in prediction performance were observed among the models with phlebitis occurrence as the binary outcome. Factors with high predictive importance included inserted site, catheter material, age, and nicardipine. Predictive models for the occurrence of PIVC-related phlebitis in orthopaedic patients have been reported [25]. However, to the best of our knowledge, there has been no report of predictive models for the occurrence of PIVC-related phlebitis in critically ill patients. Risk factors for phlebitis may vary depending on the clinical setting [7,11,14]. Therefore, it is inevitable that a prediction model of PIVC-related phlebitis occurrence should be developed for each group of target patients, such as critically ill patients, orthopaedic patients, and paediatric patients. The previous study used sophisticated statistical methods (Bayesian regression model) to create a prediction model for PIVC-related phlebitis [25], but the target population was limited to orthopaedic patients. Unlike patients managed in general wards, critically ill patients admitted to the ICU are administered several drugs, and it is necessary to consider the effect of those drugs in the prediction model. However, the previous prediction model for orthopaedic patients did not include enough drugs as risk factors, thereby making it difficult to apply the model to predict the occurrence of phlebitis in critically ill patients. In addition to the LR model that treated the outcome of phlebitis as a binary variable, this study also examined a predictive model for time-to-event outcome that considers the time until the occurrence of phlebitis. The notion of time is very important in the outcome of phlebitis, and the outcome of peripheral venous catheter complications, such as phlebitis, should be examined in terms of time-to-event outcomes [7,8,14]. To date, there have been no reports of studies using time-to-event outcomes in the development of predictive models for intravascular catheter-related complications. Therefore, our model for predicting the occurrence of phlebitis in critically ill patients can be considered novel.

Owing to a lack of predictive models for phlebitis in critically ill patients, it was unclear what type of model building method should be used to create predictive models. Bayesian regression model was used to construct a prediction model for PIVC-related phlebitis in orthopaedic patients, as mentioned above [25]. Several prediction models have also been reported to predict thrombotic complications in peripherally inserted central venous catheter (PICC) [42,43,44,45,46] and central venous catheter (CVC) [45, 47,48,49]. Moreover, LR models have also been used in most of the abovementioned studies to create prediction models. Regression models assume linearity between risk factors and outcomes. However, given that the relationship between phlebitis and risk factors is not necessarily based on linearity, creating predictive models using LR models or COX models may not be sufficient as methods for the prediction of occurrence of PIVC-related phlebitis. Considering that ML enables the construction of predictive models that do not assume linearity between exposure variables and outcomes, it has been widely used in recent years, and the fields of emergency medicine and intensive care are no exception [15,16,17,18,19,20,21,22]. The ML models mainly used in the articles reported to date are LASSO, RF, and gradient boosting decision tree [15,16,17,18,19,20,21,22,23]. In the current study, we used these three models for binary outcomes along with the RSF for time-to-event outcomes to construct prediction models [15,16,17,18,19,20,21,22, 34]. Given that each model has its own characteristics, it is important to compare which method of model building is better. The predictive performance of the previously reported models for complications of intravascular catheters was 0.716 for phlebitis in PIVC, 0.65–0.82 for venous thromboembolism in PICC [42, 44–45, 48], and 0.69–0.80 for catheter-related blood stream infection in CVC [43, 46, 49]. On the other hand, in the current study, the predictive performances with the c-statistic of the model for PIVC-related phlebitis in critically ill patients were 0.68 (95% CI 0.62–0.73) for RF and 0.66 (95% CI 0.60–0.71) for RSF. It is difficult to compare the results of this study with those of previous studies because of the differences in the patient backgrounds. The prediction performance of a clinical prediction model is considered moderate if it exceeds 0.7 [50]. The prediction performance of the model developed in this study was poor because of several factors that contributed to the development of PIVC-related complications, thus making it difficult to accurately predict the occurrence of PIVC-related outcomes. Although the number of factors used to predict outcomes is large due to the various types of drug administered in critically ill patients, there are roughly 10 non-drug predictors, and these machine learning predictive models are likely to be easy to use clinically. However, factors that influence PIVC-related complications can often be easily modified, such as PIVC insertion site and catheter gauge. Therefore, even if the predictive performance is not very high, the predictive model itself may be clinically useful because interventions can be easily implemented. Additionally, although the number of factors used to predict outcomes is large due to the various types of drugs administered in critically ill patients, there are roughly 10 non-drug predictors, and these machine learning predictive models are likely to be easy to use clinically. The benefits of creating a predictive model for development of PIVC-related phlebitis using ML includes the ability to identify high-risk patients and optimization of preventative strategies for such patients. Additionally, the model can be used to monitor the effectiveness of interventions and for the evaluation of safety and efficacy of new treatments. This can ultimately lead to better patient outcomes and reduced healthcare costs. Therefore, the predictive model itself may be clinically useful even if the predictive performance of the created model is not very high.

The benefits of creating a predictive model for development of PIVC-related phlebitis using ML includes the ability to identify high-risk patients and optimisation of preventative strategies for such patients. Additionally, the model can be used to monitor the effectiveness of interventions and for the evaluation of safety and efficacy of new treatments. This can ultimately lead to better patient outcomes and reduced healthcare costs. Furthermore, this study was able to create a predictive model for phlebitis development by considering the survival time. By evaluating the predictive models for occurrence and survival time, it was possible to create a predictive model that would be easier to use in clinical practice. Although the number of factors used to predict outcomes is large due to the various types of drug administered in critically ill patients, there are roughly 10 non-drug predictors, and these machine learning predictive models are likely to be easy to use clinically.

This study had a few limitations. First, the predictive performance of the model for phlebitis was approximately 0.7 on the AUC scale, which was not indicative of a high predictive performance. However, various factors affect the occurrence of IV catheter-related complications, not just phlebitis, and it may be difficult to achieve the same high prediction performance as other prediction models [21–22]. Although we incorporated all the previously reported risk factors for phlebitis into our model [3,4,5,6,7,8,9,10,11,12,13,14], there may still be unknown risk factors. Second, the predictive models were not validated externally. Although validation cohort were created within the same cohort by using the cross-validation method, it may not have been sufficient. External validation in a different cohort is needed to assess the generalizability and validity of the predictive models developed in this study, particularly for the random forest model which may be prone to overfitting based on the high c-statistic in the development cohort. Third, among the several factors incorporated into the predictive model in this study, the drug factor may have been inadequately handled. Medications are known to have a significant effect on the occurrence of PIVC-related phlebitis, and critically ill patients receive a variety of medications. The influence of medications on PIVC-related phlebitis may not only depend on their administration or absence but also on factors such as dosage, duration of administration, and initiation and termination times. In the prediction model created in this study, we only considered the presence or absence of medication administration and did not account for the potential effect of medications as time-dependent factors. Considering that critically ill patients are likely to receive a greater variety of medications, incorporating the influence of medications as time-dependent factors into the prediction model could potentially lead to improved predictive performance. Finally, some of the factors incorporated into the predictive model in this study, particularly those related to catheters, had high missing rates. Although multiple imputation method was used, it was not sufficient, and the predictive model may not have accurately predicted the outcomes.

## Conclusion

The RSF model for the survival time analysis of phlebitis occurrence showed relatively high prediction performance compared with the COX model. However, no significant differences in prediction performance were observed among the models with phlebitis occurrence as the binary outcome. Further investigations are required to develop models that accurately predict PIVC-related phlebitis in critically ill patients.
